# UTILIZING UNDERGRADUATE RESEARCH TO INFORM COMMUNITY RESOURCES FOR OLDER ADULTS: A SERVICE-LEARNING PROJECT

**DOI:** 10.1093/geroni/igz038.1974

**Published:** 2019-11-08

**Authors:** Amanda E Barnett, Sara Olinger

**Affiliations:** Dunn County Human Services, Menomonie, Wisconsin, United States

## Abstract

Engaging undergraduate students with aging-focused community resources is critical for preparing students to work with older adults and make positive contributions to aging societies. During the fall, 2018 semester, undergraduate students in a human development course on middle and late adulthood partnered with a county aging and disability resource center (ADRC) in Wisconsin to evaluate and update several of their existing programs and resources using empirical research. Upon completion of this project, students synthesized course material to meet all course learning objectives such as: (1) critically analyze physical, psychological, and sociological processes of aging across categories of difference (e.g. cultural, ethnic, class); (2) evaluate social policies and their multigenerational implications for midlife and older adults; and (3) construct a personal position on aging that integrates theory, research, and policy to demonstrate a sensitive and competent approach to working with midlife and older adults. Students researched, wrote reports, and presented to ADRC staff on the impact of social isolation on older adults, best practices for home visitor and transportation programs serving older adults, cognitive competency tools and best practices for utilizing memory assessments, grief supports and groups for family caregivers, and best practices for supporting veterans as they age. The outcome of these projects are research-based recommendations for any ADRC to consider when developing and implementing related programs. All stakeholders (students, professor, and ADRC staff) were satisfied with the process and outcomes of the project. Strengths and challenges of carrying out such a collaborative project will be reviewed.

